# Short- and Long-Term Reproducibility of Nighttime Blood Pressure Phenotypes and Nocturnal Blood Pressure Reduction

**DOI:** 10.1161/HYPERTENSIONAHA.120.16827

**Published:** 2021-03-22

**Authors:** Giuseppe Mancia, Rita Facchetti, Michele Bombelli, Fosca Quarti-Trevano, Cesare Cuspidi, Guido Grassi

**Affiliations:** Policlinico di Monza (G.M.), University of Milano-Bicocca, Italy.; University Milano-Bicocca, Milan (G.M.), University of Milano-Bicocca, Italy.; Clinica Medica, Department of Medicine and Surgery (R.F., M.B., F.Q.-T., C.C., G.G.), University of Milano-Bicocca, Italy.

**Keywords:** blood pressure, lacidipine, phenotypes, prognosis, risk

## Abstract

In 1722 hypertensives recruited for the European Lacidipine Study on Atherosclerosis and treated with atenolol or lacidipine (±additional drugs) during 4 years, we evaluated the long-term reproducibility of dipping, nondipping, extreme dipping, and reverse dipping, an information of key relevance for defining the prognostic impact of these blood pressure (BP) phenotypes. Ambulatory BP was measured at baseline and every year during treatment, allowing repeated classifications of these conditions. Based on systolic BP, at baseline 50.1%, 37.5 %, 4.5 %, and 7.9 % were dippers, nondippers, extreme dippers, and reverse dippers, respectively. Antihypertensive drug treatment reduced the number of dippers and extreme dippers in favor of nondippers and reverse dippers. During treatment, the dipping and nondipping phenotypes were markedly unstable and shifts from one to other phenotypes were even more common in reverse and extreme dippers In only a very small fraction of patients, a given nighttime BP phenotype was persistently found throughout the 4 years. Data were similar for diastolic BP and in subgroups differing for the type of treatment, the treatment complexity, and the achieved on-treatment BP value Poor reproducibility characterized also the absolute nighttime BP reduction as measured by the nighttime BP fall and the night/day BP ratio. Thus nighttime BP phenotypes are all largely inconsistent, their detection over the entire treatment period involving just a minute fraction of the entire patients’ cohort.

## Abstract 2

Supplemental Digital Content is available in the text.

A large number of studies has reported that the blood pressure (BP) reduction that accompanies nighttime sleep has a protective effect, that is, that the risk of cardiovascular outcomes and mortality is greater when the nighttime BP reduction from the daytime BP values is less pronounced non-dippers.^1–7^ Some of these studies have also reported that the cardiovascular risk is especially high when the nocturnal BP reduction is replaced by a nighttime BP elevation (reverse dippers) as well as when, although the results are more variable, nighttime BP falls to an even larger degree (extreme dippers),^7–15^ especially at an advanced age.^14^

Available studies on the prognostic value of nighttime BP phenotypes have one feature in common, that is, either in untreated cohorts or in cohorts of patients under antihypertensive drug treatment, the nighttime BP phenotype was identified by one ambulatory BP monitoring only, which means that no information could be made available on the consistency of any given nighttime BP phenotype over time. This is an important limitation because in the few studies that have addressed this issue, the dipping and nondipping phenotypes were found to be poorly reproducible, that is, that a noticeable fraction of dippers at the first ambulatory BP monitoring became nondippers at the second one and vice versa.^16–20^

Reproducibility of the nighttime BP phenotypes has not been thoroughly described by the available studies, however. This was the purpose of our study which made use of the data obtained in the only antihypertensive treatment trial in which ambulatory BP was measured at baseline and every year during the 4 years of a stable antihypertensive therapeutic regimen.^21,22^ This allowed us to determine the reproducibility of the dipping and nondipping phenotypes over different time intervals, including the long-term ones. It further allowed to include in the data analysis the reproducibility of the reverse and extreme dipping phenomena, the reproducibility of absolute nocturnal BP reduction, and the effect of antihypertensive treatment on nighttime BP phenotypes. On all of these issues, data are scanty.

## Methods

The database from which the present results were obtained is available from the corresponding author upon reasonable request.

ELSA (European Lacidipine Study on Atherosclerosis) was a double-blind trial aimed at comparing the effect of calcium channel blocker versus beta-blocker treatment on carotid intima-media thickness in hypertensive patients without a recent myocardial infarction, stroke, or diabetes.^21,22^ Patients had an initial sitting office diastolic BP (DBP) between 95 and 115 mm Hg and a systolic BP (SBP) between 150 and 210 mm Hg following a 4-week washout from previous treatment and were randomized to receive either lacidipine 5 mg or atenolol 50 mg once daily. After one month, lacidipine was increased to 6 mg and atenolol to 100 mg once daily, if office DBP was not <95 mm Hg with a fall of at least 5 mm Hg. Treatment could be further upgraded at visits performed 3 and 6 months after treatment initiation by addition of hydrochlorothiazide 12.5 to 25 mg once daily in absence of BP reduction at target. Drug treatment remained thereafter unchanged until the end of the study.

### BP Measurements

Office SBP was measured 3× by a mercury sphygmomanometer at the end of the washout period, that is, immediately before randomization to treatment (baseline), at monthly intervals during the initial titration period, and at 6 months intervals thereafter. The average of the 3 values was taken as the representative value for each visit. Heart rate was measured by the palpatory method after the third BP measurement. Ambulatory BP was measured for 24 hours by validated automatic devices^23^ at baseline and at yearly intervals during treatment within a week interval from office BP measurements. The monitoring began in the morning and the measuring intervals were set at 15 minutes during the daytime (6.00 am–12.00 pm) and at 20 minutes during the nighttime (0.00 am–6.00 am).^23^ Ambulatory BP recordings were analyzed centrally where artifactual values were detected and excluded according to preselected criteria.^23^ Calculation was made of the mean 24-hour, daytime, and nighttime values as well as of the corresponding heart rate values provided by the BP monitoring device.

Table S1 in the Data Supplement shows the number of valid SBP measurements for the 24 hours, daytime, and nighttime in the whole study population and in each dipping phenotype (see below) at baseline and after 1, 2, 3, and 4 years of treatment. In each dipping phenotype and for each study step (baseline and on-treatment), the percentage of valid SBP measurements was close to the expected one, that is, 90, 72, and 18 values for 24 hours, daytime and nighttime, respectively. These figures are much greater than those reported by hypertension guidelines as necessary to consider ambulatory BP monitorings technically adequate.^24^

### Other Measurements

Measurements included additional variables, that is, serum creatinine, serum total cholesterol, serum HDL (high-density lipoprotein) cholesterol, and serum triglycerides.^21,22^ Carotid intima-media thickness was assessed ultrasonographically by certified sonographers at baseline and every year during treatment, using the average of the intima-media thickness values in the far walls of the common carotid artery and the carotid bifurcation bilaterally. Cardiovascular events were adjudicated by event monitoring Committee.^21,22^

### Data Analysis

Data from the 2 treatment groups were pooled. Only patients in whom valid ambulatory BP monitorings were available on at least 2 occasions during the treatment period were considered. Patients were classified into 4 nighttime BP phenotypes according to whether, compared with the mean daytime values, nighttime mean SBP showed (1) a decrease of >10% to 19% (dippers); (2) a decrease from 10% to 0% (nondippers); (3) an increase (reverse dippers); and (4) a decrease >20% (extreme dippers).^25,26^ The same procedure was followed for nighttime BP phenotypes based on nighttime mean DBP values.^25^

Reproducibility of each dipping phenotype was assessed from the first year of treatment onward, that is, when titration to treatment effectiveness had been completed (see section on study population) and treatment was stable, using ambulatory on-treatment SBP data of year 1 versus year 2, year 1 versus year 3, year 1 versus year 4, year 2 versus year 3, and year 3 versus year 4. In patients in whom all 4 on-treatment ambulatory BP measurements were available, calculation was also made of how often each nighttime BP phenotype was present throughout the on-treatment period. Data are expressed as mean±SD or percentage. Data analysis was extended to other aspects of the reproducibility of nighttime BP phenotypes which could be suitably addressed by the ELSA trial, that is, reproducibility according to the treatment type (calcium channel blocker and beta-blocker), treatment complexity (monotherapy and combination therapy), and achieved BP value during treatment. Reproducibility was also tested for the absolute on-treatment nighttime BP reductions, which have been reported to be better than that based on BP threshold or cutoff values,^27^ also using the night/day BP ratio (average BP during the nighttime divided by average BP during the daytime) because this ratio is mentioned by guidelines as a single value that can adequately describe the day/night BP difference.^23,24^ This was done by calculation of the intraclass correlation coefficient in patients who had all the 5 ambulatory BP monitorings (one at baseline and 4 during treatment) planned for the study. The intraclass correlation coefficient values range from 0 to +1, 0 and +1 representing no and maximal reproducibility, respectively.

ANOVA (repeated measures) was used to test trends of baseline and on-treatment data. Differences between groups were tested by the *T* test or the Mann-Whitney test (mean values) and by the χ^2^ test or the Fisher exact-test percent values). A *P*<0.05 was taken as the level of significance. Data analysis was carried out by SAS software (SAS Institute, NC).

## Results

### Baseline and On-Treatment Values

Table 1 shows the baseline and on-treatment values in patients (n=1722) who had at least two on-treatment ambulatory BP measurements and thus were further analyzed for reproducibility of the nighttime BP phenotypes. BP, heart rate and carotid intima-media thickness values are shown for baseline and each year of treatment while other values are shown for baseline and the end-of-treatment. Patients were more frequently males and had an average age of <60 years. At baseline office and 24-hour, daytime, and nighttime, SBP and DBP were all elevated, whereas heart rate values were within the normal range. There was also an elevation of total serum cholesterol and carotid intima-media thickness, whereas other biochemical correlates were normal. Treatment was associated with a significant and consistent reduction of all SBP and DBP values, a heart rate reduction (due to the inclusion in the pooled data of the group treated with a beta-blocker), a modest elevation of serum triglycerides and carotid intima-media thickness, and a somewhat more pronounced increase in the incidence of type 2 diabetes. During the trial, there were 60 cardiovascular events, 12 cardiovascular deaths, and 30 all-cause deaths.

**Table 1.**
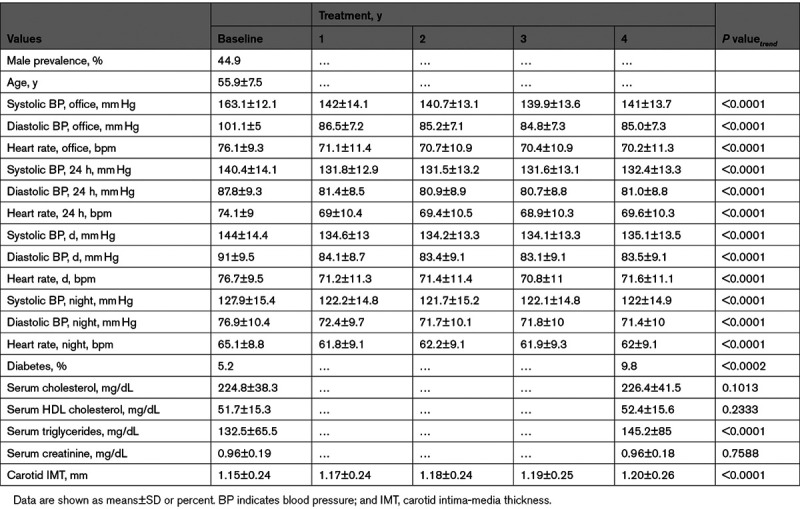
Demographic and Clinical Values of the Study Population at Baseline and During Each Year of Antihypertensive Drug Treatment

### Reproducibility of the Nighttime BP Phenotypes

**Figure 1.**
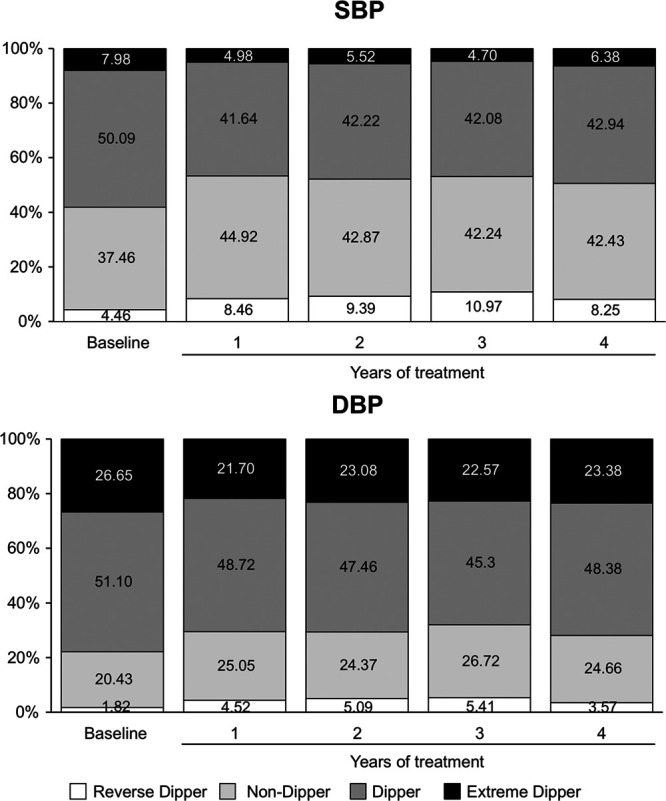
**Prevalence of different nighttime systolic blood pressure (SBP) and diastolic blood pressure (DBP) phenotypes at baseline and during the 4 y of antihypertensive treatment in 1722 patients of the ELSA (European Lacidipine Study on Atherosclerosis) trial.** For each phenotype data are shown as percentage of the data for the overall study population.

Figure 1, upper part, shows the prevalence of each nighttime BP phenotype at baseline and for each year of the 4-year treatment in the 1722 patients of Table 1. The percentage of dippers decreased from baseline to the first year of treatment and remained thereafter stable. The reverse was the case for nondippers. Extreme and reverse dippers were much rarer than dippers and nondippers, the former showing a stable decrease and the latter an increase from baseline to the on-treatment state. Similar findings were obtained for DBP (Figure 1, lower part) and for patients treated with the calcium channel blocker and the beta-blocker separately analyzed (Figure S1).

**Figure 2.**
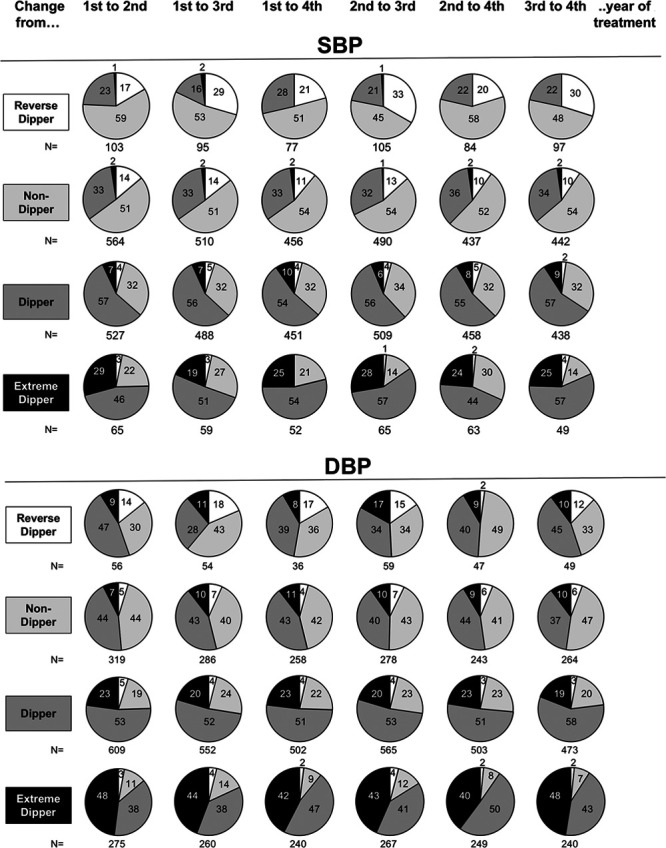
**Changes of different nighttime blood pressure (BP) phenotypes for each couple of 24-h ambulatory BP monitoring during the 4 y treatment period in the ELSA trial (European Lacidipine Study on Atherosclerosis).** For each phenotype and each couple, data refer to the percentage of patients remaining in the same phenotype or shifting to other phenotypes at the subsequent ambulatory BP monitoring. Data refer to the patients of Figure 1. Numbers at bottom of each circle refer to the size of the analyzed group. DBP indicates diastolic blood pressure; and SBP, systolic blood pressure.

**Figure 3.**
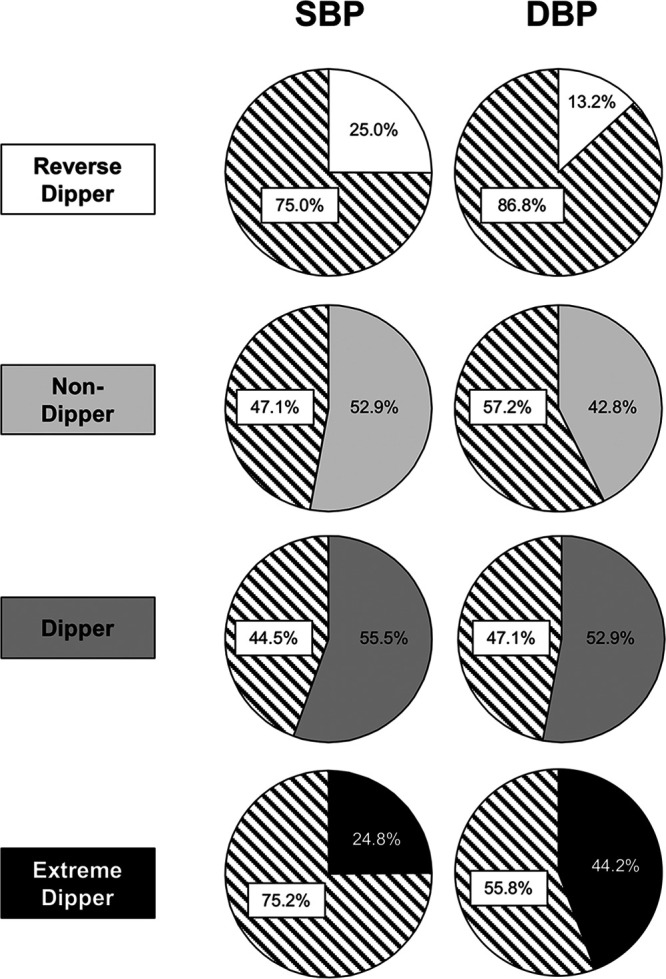
**Percentage of patients remaining in the same nighttime systolic blood pressure (SBP) or diastolic blood pressure (DBP) phenotype or shifting to another phenotype at a second ambulatory BP monitoring during the treatment period.** Data are the average of the 6 sets of ambulatory BP monitoring couples shown in Figure 2. The white part of each circle refers to the percentage of patients who shifted from the original to other nighttime BP phenotypes.

Figure 2, upper part, shows the reproducibility of each nighttime SBP phenotype when an ambulatory SBP monitoring was compared with a second monitoring at a 1- to 4-year interval. With no exception, the second nighttime BP condition exhibited major differences from the first one. More than 40% of the patients categorized as dippers at the first ambulatory BP recording changed the dipping status at the second recording, in more than 30% of the cases becoming nondippers. An even greater shift was exhibited by nondippers, >45% of whom changed the dipping phenotype at the second ambulatory BP monitoring, one-third of them becoming nondippers. Shifts were even more common in reverse and extreme dipper phenotypes who maintained the same dipping status at the second ambulatory BP monitoring in less than one-third of the cases. Similar findings were obtained for nighttime DBP-based phenotypes (Figure 2, lower part). The percentage of patients maintaining or changing the initial on-treatment nighttime BP phenotype is shown in Figure 3 as the average of the data from the 6 couples of on-treatment ambulatory BP monitorings.

**Figure 4.**
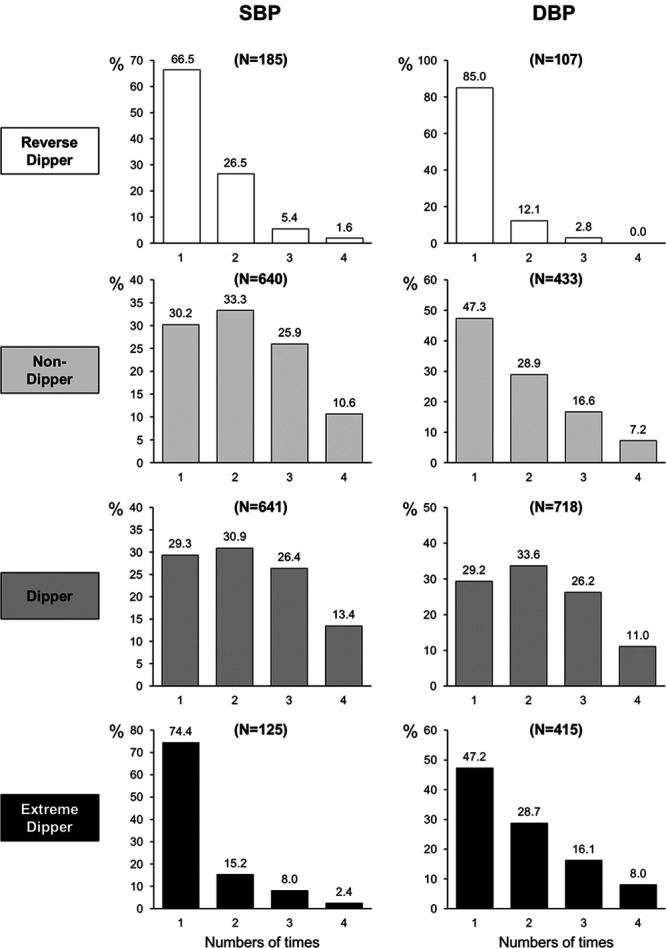
**Persistence of different nighttime systolic blood pressure (SBP) or diastolic blood pressure (DBP) phenotypes during the 4 y of antihypertensive treatment of the ELSA trial (European Lacidipine Study on Atherosclerosis) in the 815 patients in whom all yearly 24-h ambulatory BP monitorings were available.** Figures above the histograms refer to percent values (%).

Figure 4 shows the results of 815 patients in whom all on-treatment ambulatory BP monitorings were available. Regardless of whether the phenotype classification was based on SBP or DBP, in only a small fraction of the patients, a given nighttime BP phenotype was persistently found throughout the 4 years of treatment. This inconsistency was maximally evident for the extreme dipper and reverse dipper phenotypes in which a 4-year persistence of a dipping phenotype ranged from 0% to 8% of the 815 patients. There was no substantial difference in the proportion of patients persistently or less persistently exhibiting a given nighttime BP phenotype according to the type of antihypertensive treatment employed (Figure S2), the treatment complexity, that is, whether patients were on monotherapy or on combination therapy (initial drug of interest plus a diuretic; Figure S3), or the on-treatment BP value above or below the median value during the treatment period (Figure S4).

### Reproducibility of Absolute Nighttime BP Reduction

The reproducibility of the absolute nighttime BP falls in the 815 patients in whom all 4 on-treatment ambulatory BP monitorings were available are shown in Table 2. The nocturnal SBP and DBP falls were less during treatment than at baseline, their average values being similar over the 4 years of treatment. For SBP, the intraclass correlation coefficient was much lower (0.40) than the value indicating maximal reproducibility, this being the case also for DBP (0.34) and for the night-to-day BP ratio.

**Table 2.**
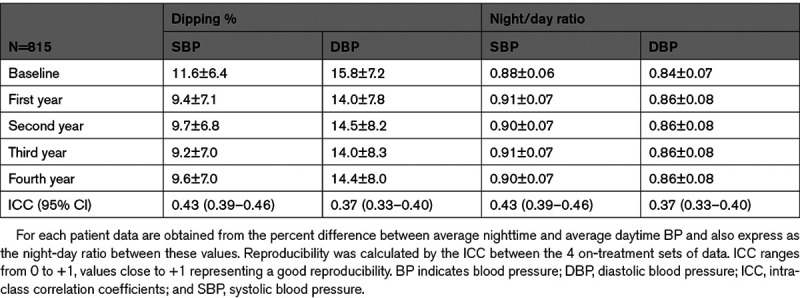
Nighttime Reduction of SBP and DBP Values (Mean±SD) at Baseline and at Each Year of Antihypertensive Treatment in the 815 Patients in Whom All 5 Ambulatory BP Monitorings Were Available

Except for the nighttime SBP values, there was no significant difference in baseline and on-treatment demographic and clinical variables between patients with maximal and minimal consistency of any given nighttime BP phenotype, that is, in patients in whom a given phenotype was detected in all or in only one on-treatment ambulatory BP monitoring (Tables S2 and S3). The latter finding included the mortality incidence (Table S3).

## Discussion

Our study shows that in treated hypertensive patients, the nighttime BP phenotypes that can be identified by 24-hour ambulatory BP monitoring have poor reproducibility. In our patients, this was the case for both the dipping and the nondipping BP phenotypes which were replicated at a second ambulatory BP monitoring in only slightly >50% of the patients. It was even more the case for less common phenotypes, such as reverse dipping and extreme dipping, which were replicated at a second ambulatory BP monitoring in <50% of the patients and only in 1 out of 4 patients when these phenotypes were established based on nighttime SBP changes. This importantly extends the conclusions of previous studies^16–20^ which were based on a much smaller number of patients and examined the nighttime BP phenotype reproducibility over one day or few weeks intervals, limited to the dipping and nondipping status. The new information provided by the present study is that (1) the results were based on a much larger number of patients; (2) poor reproducibility was shown for detection of nighttime BP phenotypes at variable time intervals, that is, from 1 to 4 years; (3) data included all nighttime BP phenotypes, that is, not only dippers and nondippers but also reverse and extreme dippers; and (4) the results were similar regarded the type of treatment, the treatment complexity, and the higher or lower BP values achieved during the treatment period.

Finally, and most importantly, because our data provided multiple ambulatory BP monitorings in a relatively large number of patients followed for 4 years, our study shows for the first time that during prolonged antihypertensive treatment all nighttime BP phenotypes are largely inconsistent, their persistence over the entire treatment period involving just a very small fraction of any nighttime phenotype group. This is relevant to the studies on the prognostic value of nighttime BP which have been based on a follow-up of several years during which data from only one ambulatory BP monitoring were available.^1–15^ Whether the results reflect the prognostic value of an occasional or a more consistent nighttime BP phenotype thus remain undetermined.

Several other results deserve to be mentioned. First, the average prevalence of the different nighttime BP phenotypes was remarkably stable throughout the entire treatment period, which means that the large number of patients who changed their original phenotype was rather precisely replaced by patients exhibiting opposite phenotype shifts. Second, in this setting, compared with baseline values, antihypertensive treatment was characterized by a reduction in the magnitude of the nocturnal BP reduction as well as in the percentage of dippers and extreme dippers in favor of nondippers and reverse dippers, indicating that the therapeutic intervention had an attenuating effect on nocturnal hypotension. The attenuation was similar in patients treated with a beta-blocker and in those treated with a calcium channel blocker, which suggests that the BP-lowering effect per se, rather than a specific influence of the drugs employed on the magnitude of nocturnal hypotension, was probably responsible. In this setting, however, an interesting observation is that the nighttime BP phenotypes characterized by greater nocturnal hypotension (dippers and extreme dippers) consistently remained the most common dipping patterns not only at baseline but also during treatment, even more so when phenotype prevalence was based on DBP. Three, the more extreme nighttime BP phenotypes, that is, reverse dipping and extreme dipping, were on average much less common than the other phenotypes both at baseline and during treatment, their detection by the ambulatory BP monitoring available in the study rarely exceeding one out of 20 patients, except for the baseline and on-treatment DBP identified extreme dipping which was detected in one out of 5 patients. The much rarer prevalence of these phenotypes explains why, in general, investigations of their relationship with BP-related outcomes have faced greater difficulties and produced somewhat inconsistent results. Finally and most importantly, the most frequent shifts of a dipping pattern involved adjacent nighttime BP phenotypes, that is, from dippers to nondippers, nondippers to dippers, extreme dippers to dippers, and reverse dippers to nondippers. However, shifts from one nighttime BP phenotype to nonadjacent ones were by no means rare, as exemplified by the relatively high percentage of patients who shifted from extreme dippers to nondippers or even reverse dippers or from reverse dippers to dippers and extreme dippers (more than one-third of all these shifts) occurring in these extreme nighttime BP. This means that poor reproducibility of nighttime BP phenotypes is not only caused by small differences in nocturnal BP reduction (1–2 mm Hg) from one ambulatory BP monitoring to another but that large BP differences (>10 mm Hg) can also occur and be responsible. This is confirmed by the evidence that poor reproducibility extended to its assessment by the absolute nocturnal SBP and DBP reductions and night-day BP ratios which exhibited an intraclass coefficient of variation well below a value indicative of good reproducibility. Other, and probably multiple, factors may thus be involved, such as (1) the imperfect identification of the beginning, the persistency, and the end of nighttime sleep even with help of the patient’s diary; (2) the disturbing effect of nighttime cuff inflations^23,28,29^; (3) the fact that, even in absence of environmental disturbances, sleep phases and depth may rarely be superimposable night after night^30,31^; and (4) a variable adherence to the treatment prescribed between one ambulatory BP recording and another. This is an unsurmountable limitation in absence of the comparison provided by electroencephalographic, eye measurement, and myographic recordings, which, however, are hardly a feasible approach in an outpatient setting.

## Perspectives

Our study has several elements of strength but also weaknesses. The strengths are (1) the relatively large number of patients compared with previous studies, (2) the consistency of the reproducibility data on each nighttime BP phenotype across many different couples of ambulatory BP monitoring, and (3) the quality of ambulatory BP monitoring, including the nighttime period, as shown by the high number of valid BP readings throughout the day and night. This is a limitation of previous studies on nocturnal hypotension in which information on the quality and number of nocturnal BP readings as well as of their homogeneity between patients is not regularly reported. The weaknesses are that we were unable to provide mechanistic data for the poor reproducibility of nighttime BP phenotypes between different ambulatory BP monitorings. Furthermore, we could not identify any baseline or on-treatment predictive marker of the greater or lesser stability of the nighttime BP phenotypes. And finally, we could also not address the possible prognostic differences of more versus less consistent nighttime BP phenotypes because, as shown in Table S2, in the ELSA trial, the number of events was small and not suitable for any meaningful data analysis. Nor could outcome data be replaced by a surrogate clinical measure such as carotid intima-media thickness because prediction of cardiovascular outcomes by carotid intima-media thickness has been reported for absolute baseline pretreatment values^32,33^ but not for treatment-related changes.^34,35^ The predictive value of persistent versus occasional nighttime BP phenotypes remains therefore an important issue to be addressed by future studies in which serial ambulatory BP monitorings and outcome data will be available.

## Sources of Funding

None.

## Disclosures

None.

## Supplementary Material



## Novelty and Significance

### What Is New?

- The present investigation, based on the analysis of the data collected in the ELSA trial, was aimed at providing information on the long-term reproducibility of nighttime blood pressure (BP) phenotypes as well as on the effects of antihypertensive treatment on these phenotypes.
- Information was obtained in 1772 hypertensive patients in whom 24-hour ambulatory BP was measured before and at last twice (yearly measurements) during a 4-year antihypertensive treatment based on a beta-blocking drug or a calcium channel blocker.

### What Is Relevant?

- The study provides evidence that in treated hypertensive patients all nighttime BP phenotypes that can be identified by 24-hour BP monitoring have a poor reproducibility.
- This was the case for the dipping and the nondipping BP phenotypes which were replicated at a second ambulatory BP monitoring in about 50% of the patients only. It was even more the case for less common phenotypes, such as reverse dipping and extreme dipping, replicated at a second 24-hour BP monitoring in <50% of the patients. In the 815 patients in whom ambulatory BP monitoring was done yearly over the 4-year treatment, any given nighttime BP phenotype was consistently detected in only a very small fraction of the patients.
- Poor reproducibility also extended to absolute nocturnal BP reduction and to the night-day BP difference.
- During antihypertensive treatment, there was a reduction in the number of dippers and extreme dippers in favor of nondippers and reverse dippers, indicating that the therapeutic intervention had an attenuating effect on nocturnal hypotension.

### Summary

The present study performed in the largest population sample ever examined shows for the first time that reproducibility of different nighttime BP patterns (dipping, nondipping, extreme, and reverse dipping) is extremely limited. This finding is relevant to the studies on the prognostic value of nighttime BP which have been based on a follow-up of several years during which data from only one ambulatory BP monitoring were available.
