# Supplementary material for: Short- and Long-Term Reproducibility of Nighttime Blood Pressure Phenotypes and Nocturnal Blood Pressure Reduction
Source: Hypertension. 2021 Mar 22;77(5):1745–55. doi: 10.1161/HYPERTENSIONAHA.120.16827 (PMC9634725; doi:10.1161/HYPERTENSIONAHA.120.16827)

SUPPLEMENTAL DATA.

SHORT- AND LONG-TERM REPRODUCIBILITY OF NIGHTTIME BLOOD PRESSURE PHENOTYPES AND NOCTURNAL BP REDUCTION

Giuseppe Mancia[1,2], Rita Facchetti[3], Michele Bombelli[3],

Fosca Quarti-Trevano[3], Cesare Cuspidi[3] and Guido Grassi[3].

[1]Policlinico di Monza, [2]University Milano-Bicocca, Milan, [3]Clinica Medica, Department of Medicine and Surgery, University of Milano-Bicocca, Italy.

Running title: *Blood Pressure Phenotypes*

Words count*:* 5915
Words Abstract: 223
Figures: 4
Tables: 2
Supplementary Tables: 3
Supplementary Figures: 4

Corresponding Author*:*
Prof. Giuseppe Mancia
University of Milano-Bicocca
Piazza dei Daini 4, 20126 Milano, Italy
Tel: 0039 3474327142
e-mail: giuseppe.mancia@unimib.it

Table S1. Number of valid BP readings over the 24 hours, the daytime and the nighttime in all patients and in the different nighttime BP phenotypes classified by nighttime SBP changes compared to daytime values. Based on the intermittency of the automatic BP readings and the duration of the day and night periods (18 and 6 hours) the expected number of BP values for each ambulatory BP monitoring were 90 for the 24 hours, 72 for the daytime and 18 for the nighttime.

			Number of measurements		
**Patients**	**Visit**		**24h**	**Daytime**	**Nighttime**
**All Patients**	Baseline		79.0±13.1	61.3±11.2	17.8±4.4
	Treatment (Years)				
		1	77.2±14.6	59.5±12.2	17.9±4.8
		2	78.9±13.1	61.9±10.8	17.3±3.2
		3	78.5±13.1	62.0±10.6	16.9±3.0
		4	78.9±13.1	62.3±10.6	17.0±2.7
**Nighttime BP phenotype**					
Reverse Dippers (N=71)	Baseline		72.1±16.7	55.6±13.9	16.5±5.8
	Treatment (Years)				
		1	69.8±17.9	53.1±14.8	16.9±6
		2	73.7±17.9	57.6±13.8	16.7±4.3
		3	73.7±15.6	58.4±11.7	15.8±4.1
		4	75.3±13.2	58.9±10.8	16.4±3.9
Non Dippers (N=596)	Baseline		77.7±13.2	60.2±11.1	17.5±4.7
	Treatment (Years)				
		1	76.1±15.1	58.7±12.3	17.5±5.0
		2	78.5±12.5	61.5±10.2	17.3±3.3
		3	77.8±13.7	61.2±11.1	16.9±3.2
		4	78.8±12.8	62.1±10.4	17.1±2.5
Dippers (N=797)	Baseline		80.6±11.5	62.6±10.3	18.1±3.9
	Treatment (Years)				
		1	79.1±13.3	61.3±11.4	18.0±4.4
		2	79.9±12.7	62.7±10.5	17.4±3.2
		3	80.1±11.4	63.3±9.2	17.0±2.6
		4	79.9±12.5	63.1±10.3	17.1±2.7
Extreme Dippers (N=127)	Baseline		81.1±11.5	63.4±10.0	17.7±4.0
	Treatment (years)				
		1	78.6±13.7	61.2±11.3	17.7±4.3
		2	80.7±11.8	63.7±10.2	17.4±2.3
		3	79.7±13.3	63±10.9	17.0±2.1
		4	80.1±12.9	63.3±10.6	17.1±2.2

Data are shown as means±standard deviations.BP:blood pressure,SBP:systolic blood pressure

Table S2. Baseline BP, heart rate and other variables showing minimal and maximal persistance of a given nighttime SBP phenotype, i.e. from one to four times over the four years of antihypertensive treatment.

**Variable**	**Numbers of times**		***p-value***	**Numbers of times**		***p-value***
	**1**	**4**		**1**	**4**	
	**Reverse-Dippers**			**Non-Dippers**		
N	123	3		193	68	
Male prevalence (%)	46.3	100	*0.1407*	48.2	36.8	*0.1037*
Age (years)	58.2±7.4	61±12.1	*0.5268*	55.4±7.2	56.2±7.7	*0.4115*
Systolic BP, office (mmHg)	165±11.8	169.7±10.3	*0.4983*	162.4±11.3	160.9±10.5	*0.3290*
Diastolic BP, office (mmHg)	101.1±4.7	99.7±2.5	*0.6047*	100.9±4.4	100.6±4.4	*0.6505*
Heart rate, office (b/min)	75.2±9.3	89.3±23.4	*0.4065*	76.4±9.5	76.4±9.9	*0.9722*
Systolic BP, 24-hour (mmHg)	142.2±14.4	140.3±17.1	*0.8233*	140±14.3	140.7±12.1	*0.6886*
Diastolic BP, 24-hour (mmHg)	88.6±10.4	80.8±10.4	*0.2000*	87±9.3	89.1±9.2	*0.0971*
Heart rate, 24-hour (b/min)	73.1±8.6	80.3±18.9	*0.5765*	74.2±9.1	74.1±10.1	*0.9445*
Systolic BP, daytime (mmHg)	145.3±14.7	143.1±19.9	*0.7954*	144.3±14.5	143.6±12.2	*0.7125*
Diastolic BP, daytime (mmHg)	91.7±10.5	82.7±11.2	*0.1422*	90.5±9.5	91.7±9.2	*0.3658*
Heart rate, daytime (b/min)	75.7±9.1	83.9±21.2	*0.5727*	76.8±9.6	76.6±10.7	*0.9225*
Systolic BP, nighttime (mmHg)	132.4±15.1	133.3±12.7	*0.9176*	125.1±15	130.6±13.2	*0.0090*
Diastolic BP, nighttime (mmHg)	79.2±10.9	75.2±10.2	*0.5271*	74.9±9.9	79.7±10.1	*0.0008*
Heart rate, nighttime (b/min)	64.8±8.5	69.8±15.4	*0.3213*	65.7±9.2	65±9	*0.5733*
Diabetes (%)	8.2	0	*1.0000*	3.1	4.4	*0.7002*
Serum cholesterol (mg/dl)	223.1±39.5	236.5±20.6	*0.5630*	222.9±39.3	221.8±32.1	*0.8384*
Serum HDL cholesterol (mg/dl)	49.9±14.7	49.9±9.6	*0.9998*	51.4±17.2	50.5±14.5	*0.7176*
Serum triglycerides (mg/dl)	140.8±76.8	130.2±45.1	*0.8116*	133.9±70.7	116±47.5	*0.0541*
Serum creatinine (mg/dl)	0.95±0.2	0.84±0.17	*0.3446*	0.95±0.19	0.99±0.18	*0.1061*
Carotid IMT (mm)	1.22±0.25	1±0.06	*0.1407*	1.13±0.21	1.19±0.25	*0.0516*
	**Dippers**			**Extreme Dippers**		
N	188	86		93	3	
Male prevalence (%)	52.7	40.7	*0.0698*	50.5	33.3	*1.0000*
Age (years)	57.3±7.8	54.9±7.3	*0.0162*	55.9±6.9	52.3±1.5	*0.3775*
Systolic BP, office (mmHg)	163.6±12.1	164.2±10.7	*0.6628*	162.5±12.6	162±10.4	*0.9479*
Diastolic BP, office (mmHg)	100.3±4.5	100.7±4.7	*0.5666*	101±5.5	101.3±6.7	*0.9159*
Heart rate, office (b/min)	76.7±8.9	75.1±8.1	*0.1433*	75.3±8.7	76.7±1.2	*0.7799*
Systolic BP, 24-hour (mmHg)	139.9±13.5	139.3±14.3	*0.7427*	139.5±14.1	132.5±8.3	*0.3982*
Diastolic BP, 24-hour (mmHg)	87±9.3	87.5±11	*0.6739*	86.1±8.3	86.2±7.4	*0.9754*
Heart rate, 24-hour (b/min)	74.4±8.5	74.3±8.6	*0.9247*	73.6±8.5§	77.6±16.6	*0.4395*
Systolic BP, daytime (mmHg)	143.1±14	143.7±14.7	*0.7330*	144.4±15	138.7±7.7	*0.5192*
Diastolic BP, daytime (mmHg)	89.8±9.6	91±11.1	*0.3613*	89.9±8.7	91.4±6.6	*0.7716*
Heart rate, daytime (b/min)	77±8.9	76.9±9	*0.9333*	76.1±8.9§	80.6±16.9	*0.4050*
Systolic BP, nighttime (mmHg)	128.8±13.9	123.4±15.5	*0.0039*	122.3±13.6	109±13.2	*0.0981*
Diastolic BP, nighttime (mmHg)	77.2±10.2	74.8±12.4	*0.0859*	72.8±8.6	66.6±10.1	*0.2274*
Heart rate, nighttime (b/min)	65.7±8.4	65±8.4	*0.5814*	64.6±9.1	66.1±15.5	*0.7924*
Diabetes (%)	4.8	1.2	*0.1792*	1.1	0	*1.0000*
Serum cholesterol (mg/dl)	231.6±37.1	222.3±39.7	*0.0610*	228.3±38.1	234.9±38.1	*0.7693*
Serum HDL cholesterol (mg/dl)	52±14.5	52.4±16	*0.8371*	54.4±19	49.2±10.7	*0.6361*
Serum triglycerides (mg/dl)	137.8±68.7	132.5±78.4	*0.5770*	131.5±59.5	114.2±53.6	*0.6199*
Serum creatinine (mg/dl)	0.95±0.19	0.94±0.19	*0.8503*	0.95±0.2	0.74±0.15	*0.0843*
Carotid IMT (mm)	1.15±0.23	1.15±0.23	*0.8582*	1.15±0.22	1.09±0.05	*0.6148*

Dta are shown as means±standard deviations. BP: blood pressure. IMT: intima-media thickness.

Table S3. BP, heart rate and other variables in patients showing minimal and maximal persistance of a given nighttime BP phenotype, i.e. from one to four times during the treatment period. Data refer to the average of the 4 measurements obtained during the 4 years of antihypertensive treatment.

**Variable - ON-TREATMENT**	**Numbers of times**			**Numbers of times**		
	**1**	**4**	***p-value***	**1**	**4**	***p-value***
	**Reverse Dippers**			**Non- Dippers**		
N	123	3		193	68	
Male prevalence (%)	46.3	100	*0.1051*	48.2	36.8	*0.1198*
Age (years)	58.2±7.4	61±12.1	*0.5268*	55.4±7.2	56.2±7.7	*0.4115*
Systolic BP, office (mmHg)	142.9±10.5	154.5±10.7	*0.0587*	141.2±10	140.5±9.4	*0.6051*
Diastolic BP, office (mmHg)	85.9±5.2	85.5±1.9	*0.8810*	85.6±5	85.1±5	*0.4934*
Heart rate, office (b/min)	70.5±8.6	68.2±3.5	*0.6462*	70.2±8.2	70.6±9	*0.7068*
Systolic BP, 24-hour (mmHg)	134.3±11.3	129.4±11.8	*0.4546*	130.8±11.8	132.4±11.2	*0.3275*
Diastolic BP, 24-hour (mmHg)	82.2±8.7	74.4±6.3	*0.1267*	80±7.7	82.2±8.2	*0.0455*
Heart rate, 24-hour (b/min)	68.7±9.2	69.2±12	*0.9214*	68.8±9.2	69.1±9.8	*0.8464*
Systolic BP, daytime (mmHg)	135.8±11.3	127.8±11.3	*0.2312*	134.1±11.8	134±11.2	*0.9764*
Diastolic BP, daytime (mmHg)	84.1±8.8	74.4±5.7	*0.0606*	83±7.9	84.1±8.2	*0.3286*
Heart rate, daytime (b/min)	70.7±9.9	70.4±11.2	*0.9654*	70.8±9.9	71.2±10.5	*0.7768*
Systolic BP, nighttime (mmHg)	129.3±11.7	134.8±13.9	*0.4213*	118.8±12.1	126.5±11.1	<*.0001*
Diastolic BP, nighttime (mmHg)	75.8±8.5	74.6±8.3	*0.8065*	69.5±7.7	75.8±8.5	<*.0001*
Heart rate, nighttime (b/min)	61.7±7.7	64.5±15.1	*0.7785*	61.8±7.8	61.7±8	*0.8932*
Diabetes (%)	15.4	33.3	*1.0000*	11	6.3	*0.7002*
Serum cholesterol (mg/dl)	224.3±41	241.5±12	*0.5558*	227.3±40.5	222.2±38.4	*0.3740*
Serum HDL cholesterol (mg/dl)	51.8±13.8	52±11.3	*0.9812*	52.6±15.5	54.5±15.1	*0.4282*
Serum triglycerides (mg/dl)	159.1±100.4	167±108.9	*0.9127*	148.2±85.5	121.9±57.9	*0.0071*
Serum creatinine (mg/dl)	0.97±0.19	0.89±0.12	*0.5324*	0.95±0.17	0.97±0.16	*0.2843*
Carotid IMT (mm)	1.25±0.23	1.15±0.15	*0.4592*	1.17±0.22	1.22±0.23	*0.1347*
	**Dippers**			**Extreme Dippers**		
N	188	86		93	3	
Male prevalence (%)	52.7	40.7	*0.0660*	50.5	33.3	*1.0000*
Age (years)	57.3±7.8	54.9±7.3	*0.0162*	55.9±6.9	52.3±1.5	*0.3775*
Systolic BP, office (mmHg)	141.5±10.3	144±9.9	*0.0519*	141.4±11.7	144±1.8	*0.6979*
Diastolic BP, office (mmHg)	84.8±4.7	86.3±5	*0.0146*	85.3±5.1	87.5±3.7	*0.4511*
Heart rate, office (b/min)	71±8.3	70.8±8.8	*0.8448*	70.5±8.5	65.8±6.5	*0.3504*
Systolic BP, 24-hour (mmHg)	131.8±11.1	130.5±11.3	*0.3880*	130±10.5	124.2±6.2	*0.3403*
Diastolic BP, 24-hour (mmHg)	80.3±7.9	80.5±9	*0.8708*	78.8±6.5	80.3±4.2	*0.6946*
Heart rate, 24-hour (b/min)	69.4±8.5	69.5±8.9	*0.8794*	68.1±8.8	66.4±14.9	*0.7518*
Systolic BP, daytime (mmHg)	134±11.5	134.7±11.6	*0.6751*	134.3±10.6	131.2±7.3	*0.6075*
Diastolic BP, daytime (mmHg)	82.7±8.1	83.8±9.2	*0.2892*	82.3±6.7	85.8±5.1	*0.3696*
Heart rate, daytime (b/min)	71.5±9.2	71.7±9.8	*0.8388*	70±9.5	68.1±14.9	*0.7389*
Systolic BP, nighttime (mmHg)	123.7±10.9	115.3±10.2	<*.0001*	114.1±9.9	97.6±3.8	*0.0051*
Diastolic BP, nighttime (mmHg)	72.2±8.2	68.3±8.6	*0.0005*	66±6	59.2±1.7	*0.0525*
Heart rate, nighttime (b/min)	62±7.4	61.7±7.6	*0.7073*	61.1±7.2	60±15.4	*0.7980*
Diabetes (%)	12.4	10.5	*0.1792*	9.2	0	*1.0000*
Serum cholesterol (mg/dl)	232.3±42.9	224.3±34.1	*0.1491*	234.6±42.2	235.3±17.8	*0.9764*
Serum HDL cholesterol (mg/dl)	52.6±16.1	54.7±18.1	*0.3791*	55.2±16.1	44.2±10.6	*0.2438*
Serum triglycerides (mg/dl)	150.7±86.6	136.8±79	*0.2232*	141.8±70.8	174.6±74.8	*0.4329*
Serum creatinine (mg/dl)	0.94±0.2	0.94±0.17	*0.8666*	0.95±0.19	0.7±0.07	*0.0245*
Carotid IMT (mm)	1.19±0.22	1.17±0.22	*0.4601*	1.2±0.24	1.12±0.1	*0.5998*

Data are shown as means±standard deviations. Abbreviations as in the precedin

Figure S1. Average number of different nighttime BP phenotypes at baseline and during the four years of antihypertensive treatment according to the type of treatment employed, i.e. the beta blocker atenolol or the calcium channel blocker lacidipine. Explanations as in preceding figures.

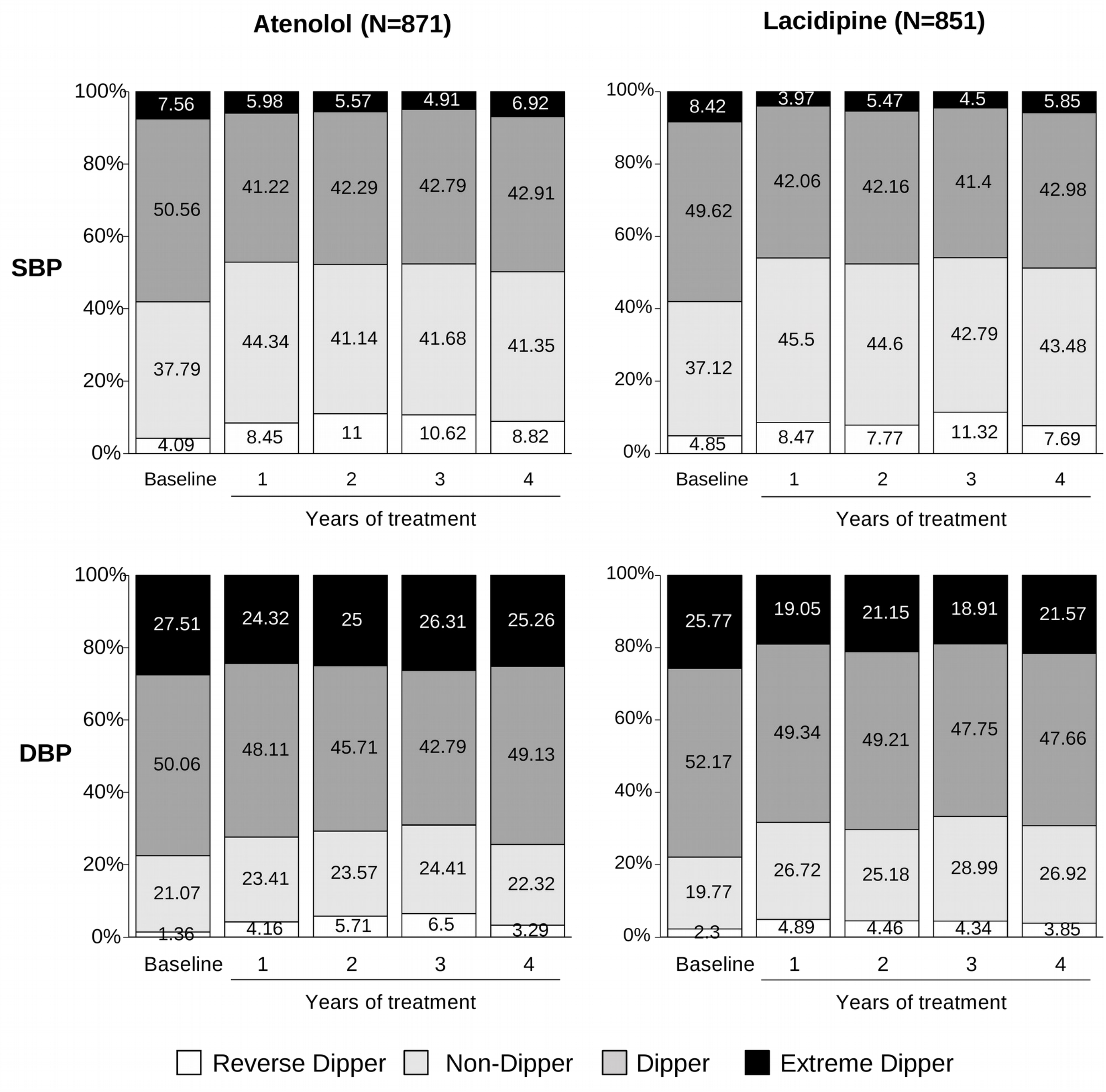

Figure S2. Persistence of different nighttime SBP or DBP phenotypes during the four years of antihypertensive treatment of the ELSA trial in 815 patients of Figure 4, according to the treatment type, beta-blocker or calcium channel blocker. Explanations as in preceding figures.

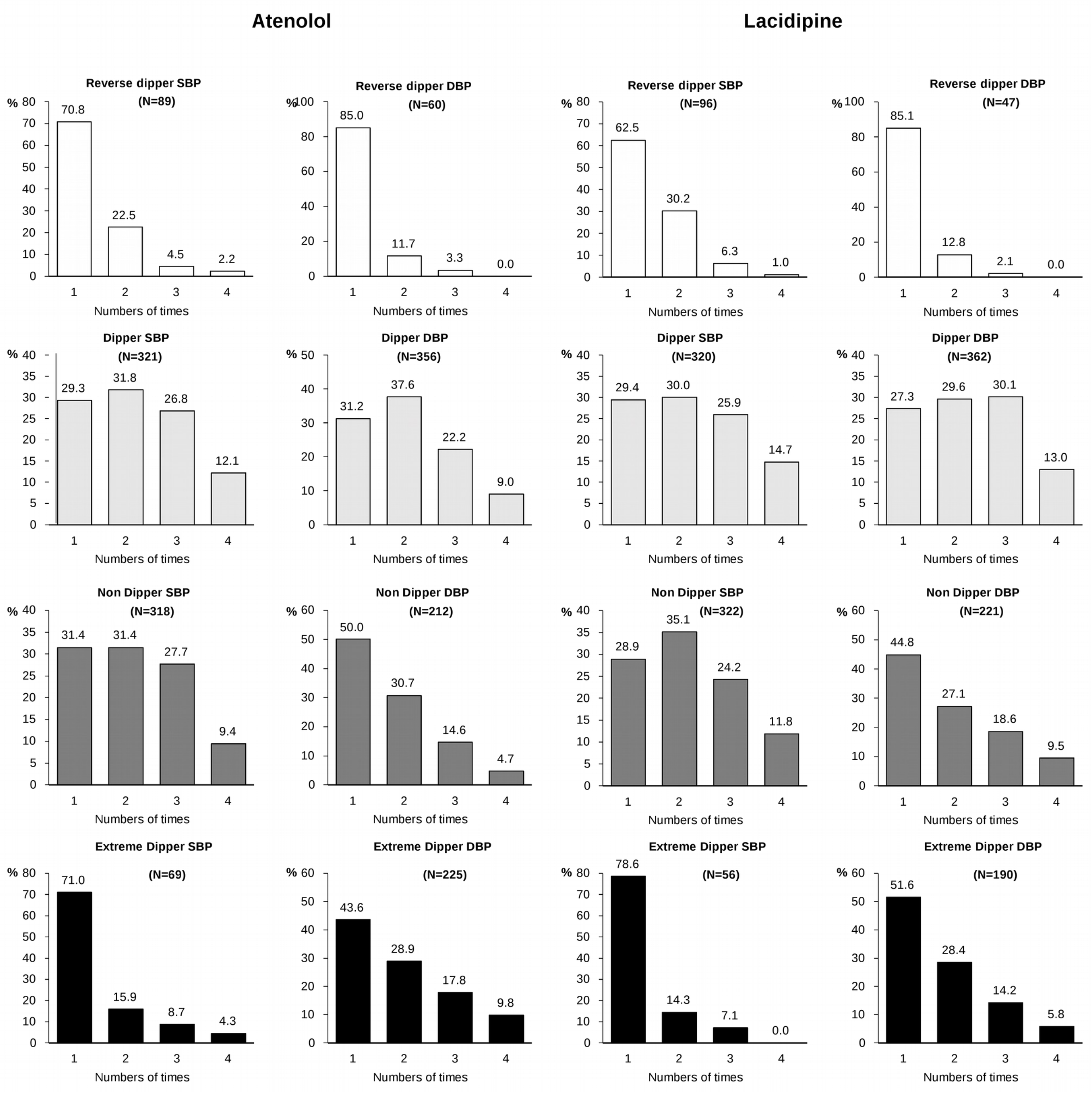

Figure S3. Persistence of different nighttime SBP or DBP phenotypes according to complexity of antihypertensive treatment, i.e. monotherapy or combination treatment (initial drug plus hydrochlorothiazide). Other explanations as in preceding figures.

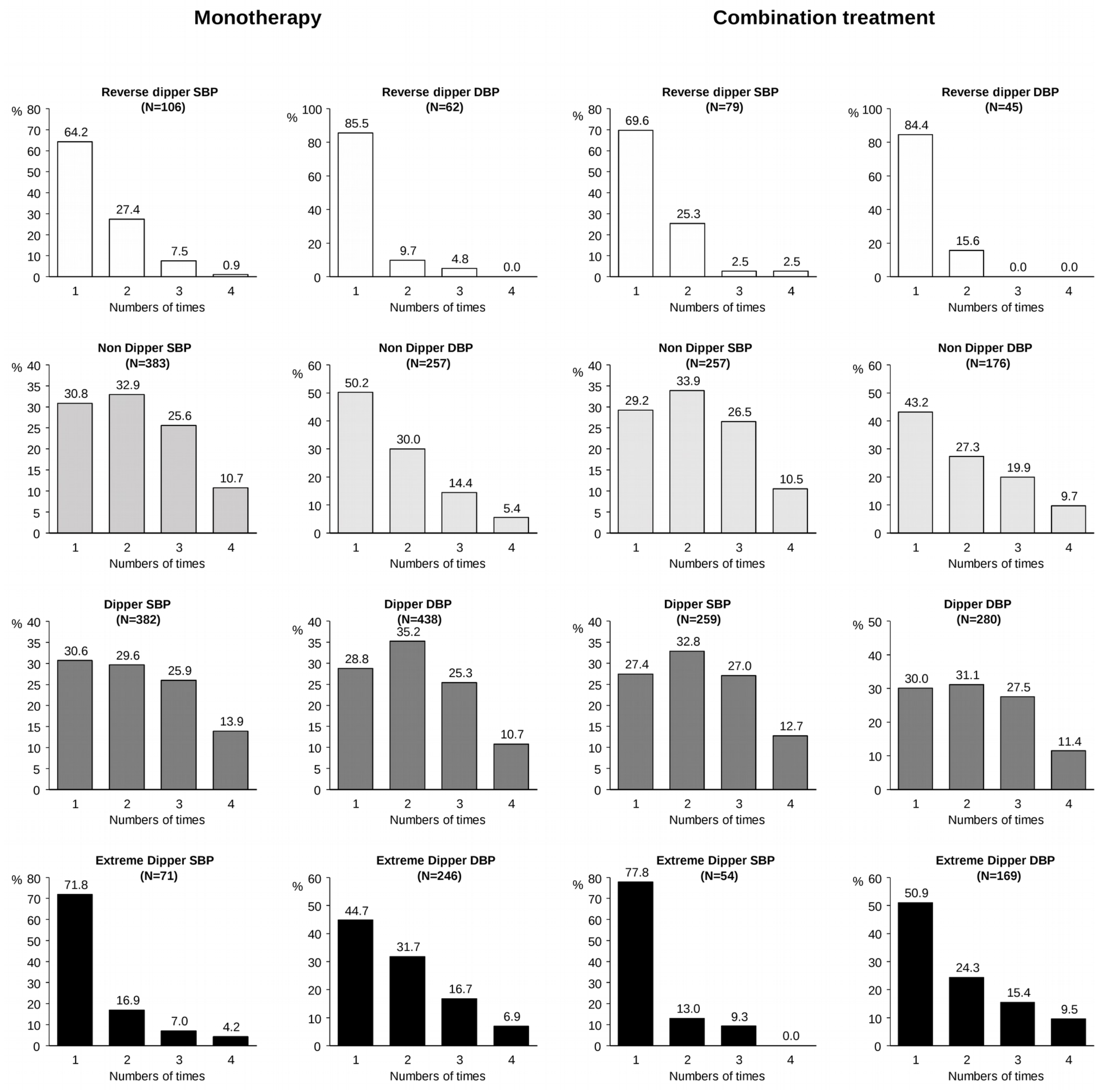

Figure S4. Persistence of different nighttime SBP or DBP phenotypes according to achieved SBP or DBP treatment-dependent reduction < or -> the 24 h BP median reduction. On-treatment 24h BP reduction was calculated as the average of the values obtained by all available on-treatment vs baseline BP values. Explanations as in the preceding figures.

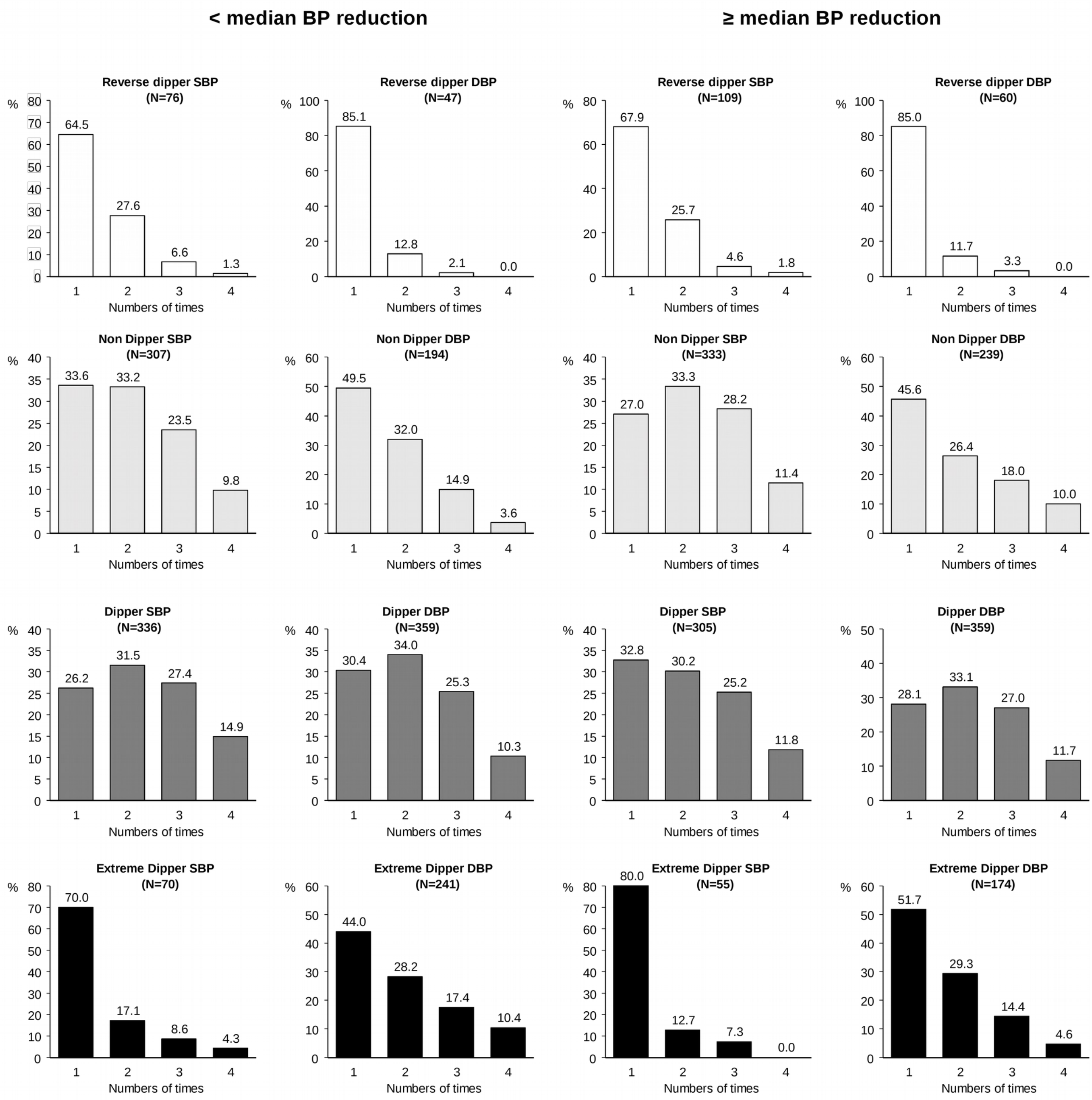

Supplement: Supplementary file 1 [file hyp-77-1745-s001.pdf]
